# Histopathology and *ARID1A* Expression in Endometriosis-Associated Ovarian Carcinoma (EAOC) Carcinogenesis Model with Endometrial Autoimplantation and DMBA Induction

**DOI:** 10.31557/APJCP.2021.22.2.553

**Published:** 2021-02

**Authors:** Puspita Eka Wuyung, Familia Bella Rahadiati, Hartono Tjahjadi, Salinah Salinah, Kusmardi Kusmardi, Ria Kodariah, Budi Wiweko

**Affiliations:** 1 *Department of Anatomical Pathology, Faculty of Medicine Universitas Indonesia. *; 2 *Animal Research Facilities, Indonesian Medical Education and Research Institute, Faculty of Medicine Universitas Indonesia.*; 3 *Specialty Programme in Anatomical Pathology, Department of Anatomical Pathology, Faculty of Medicine Universitas Indonesia. *; 4 *Department of Obstetrics and Gynecology, Faculty of Medicine Universitas Indonesia. *; 5 *Human Reproduction, Infertility, and Family Planning, Indonesian Medical Education and Research Institute, Faculty of Medicine Universitas Indonesia. *

**Keywords:** Endometriosis, EAOC, experimental animal model, DMBA, ARID1A

## Abstract

**Background:**

Ovarian carcinoma is one of the most deadly malignancies in the gynecologic field. The cause is not yet known, and the clinical symptoms are not specific. Endometrioid carcinoma and ovarian clear cell carcinoma can originate from endometriosis and are known as endometriosis-related ovarian carcinoma (EAOC). Development of EAOC experimental animal models is needed for basic research and clinical preparation of human tissue tests. This study aimed to determine the role of the *ARID1A* gene mutation in the carcinogenetic process of EAOC in experimental animal models induced with DMBA.

**Methods:**

In this study, the EAOC experimental model was developed using the autoimplantation technique and DMBA induction. This study involved placebo surgery mice (sham), endometrial autoimplantation, and a combination of endometrial autoimplantation and DMBA induction, which were sacrificed at weeks 5, 10, and 20, respectively. Histopathological assessment and immunohistochemical ARID1A staining with an assessment of positive percentages were carried out on 200 cells.

**Results:**

This study produced 1 (20%) atypical endometriosis and 1 (20%) clear cell carcinoma at implantation and after 10 weeks of DMBA induction, and 100% endometrioid carcinoma in the DMBA-induced group. ARID1A staining did not show any significant difference (p = 0.313) in all groups.

**Conclusion:**

The combination of endometrial autoimplantation techniques and DMBA induction in the ovary produced atypical endometriosis, clear cell carcinoma, and endometrioid carcinoma, where time is an important factor. There was no significant difference in ARID1A expression between the treatment and control groups.

## Introduction

Ovarian malignancy is one of the most deadly types of malignancy in gynecology with a 5-year survival rate of less than 40% (Bray et al., 2018; Mangili et al., 2012; Reid et al., 2017). GLOBOCAN data in 2018 revealed that the incidence of ovarian malignancy ranks seventh in women, with 780,000 deaths worldwide. According to the IAPI Cancer Registry, 1,351 cases of ovarian malignancy were recorded in 2013. It ranked 7^th^ in the primary organ tumors and is the 3rd most common malignancy in women (Badan Registrasi Kanker Perhimpunan Dokter Spesialis Patologi Indonesia, 2017).

Ovarian malignancy comes from three cell types, namely epithelial, stromal, and germinal cells. Epithelial malignancy is recorded in more than 90% in all ovarian malignancies. Clear cell ovarian carcinoma (CCOC) and endometrioid ovarian carcinoma (EOC) are often associated with benign endometriotic lesions. (Ellenson LH et al., 2014; Gilks et al., 2014; Kurman and Shih, 2010)

Endometriosis is a gynecological disorder that is often found in women of reproductive age. Approximately 1% of endometriosis has the potential to be malignant (Esmaili et al., 2016; Xiao et al., 2012). Ovarian malignancy associated with endometriosis is known as endometriosis-associated ovarian carcinoma (EAOC). Predisposing factors that cause endometriosis to turn into ovarian malignancy are known to involve many factors including genetic factors and epigenetic factors and/or effects of the tumor microenvironment (Akbarzadeh-Jahromi et al., 2015)

Ovarian carcinoma and adjacent endometriosis lesions have similar genetic changes (Ness, 2003). Wiegand et al., (2010) found mutations of the *ARID1A *tumor suppressor gene in endometrioid carcinoma and clear ovarian cell carcinoma. The same mutation was found in atypical endometriosis associated with ovarian carcinoma, so it was estimated that *ARID1A* mutation occurs at the beginning of the neoplastic transformation of endometriosis. Research on the transformation of malignancies in endometriosis is of current interest. This study was conducted to determine the role of the *ARID1A *gene mutation in the carcinogenetic process of EAOC in experimental animal models induced with DMBA.

## Materials and Methods

This study was carried out with an experimental design. Ethical approval was given by the ethics committee of the Faculty of Medicine, Universitas Indonesia, under file number 1028/UN2.F1/ETIK/2017. Twenty-five rats were used from the Center for Research and Development of Biomedical and Basic Health Technology, Indonesian Ministry of Health, which were divided into 5 treatment groups: endometrial autoimplantation group without DMBA induction, a combination of autoimplantation and DMBA induction techniques. DMBA (Sigma Chemical Co., St. Louis, MO) was heated to 124°C to reach the fusion point. A 3.0 silk thread with a length of 0.5 cm was immersed in the melted DMBA. Silk thread would then contain 1 mg of DMBA.

The surgery was carried out at IMERI FKUI, 3 days after acclimatization. Anesthesia was carried out with ketamine hydrochloride (73 mg/BW) and xylazine (8.8 mg/BW) intraperitoneally. Before surgery, the aseptic procedure was performed on the abdominal area using povidone iodine, and a vertical incision was made in the middle of the abdomen. Left uterine tissue was cut 1 cm long and reversed so that the endometrium appeared on the outer surface, and tissue was then placed in a cold 0.9% NaCl solution. After that, the endometrial tissue and DMBA-coated threads were implanted in the right ovary using nylon threads sized 4.0. Ceftriaxon was then administered at 20 mg/KgBB for 3 days intraperitoneally. At 5, 10, and 20 weeks, euthanasia using ketamine was performed together with the sham group, and then the implanted tissue in the right ovary was taken and fixed with 10% formalin buffer. It was then made into Hematoxylin-Eosin (HE) preparations and immunohistochemical staining was performed using ARID1A-PSG3 Santa Cruz biotechnology primary antibodies and seen under a light microscope.

Assessment of *ARID1A* expression was carried out in normal endometrial epithelial cells, epithelial and glandular cells of the autoimplanted endometriosis, and in induced tumor cells. The loss of *ARID1A* expression was assessed by loss of expression in the cell nucleus. Semiquantitative assessment using Image G refers to the study by (Fadare et al., 2012), which relies the assessment on the percentage of stained cells regardless of the intensity of the staining. The assessment was carried out on 200 cells. The data obtained were then analyzed statistically with the SPSS 22.

## Results

**Figure 1:**
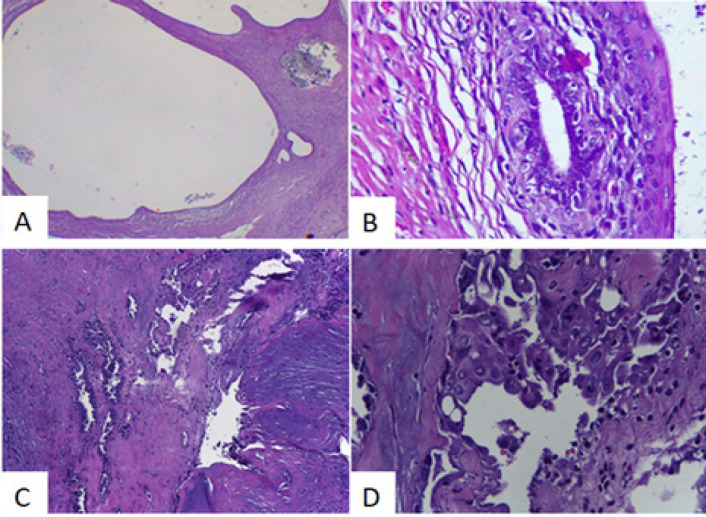
A. Endometrial cyst (HE40x). B. Endometrial cyst with squamous metaplasia (HE400x). C. Cyst with fibrotic wall, with glands lined with atypical cells in between (HE 100x). B. Stratified epithelium, tufting, and nuclear atypia (HE 400x)

In the endometrial autoimplantation group without DMBA induction, the graft tissue proliferated to resemble endometriosis in humans, in which no atypia was found. In the group with combination of autoimplantation technique and 5-week DMBA induction, graft tissue grew to resemble non-atypical endometriosis in humans, 1 of which had squamous metaplasia. In the group with combination of autoimplantation technique and 10-week DMBA induction, 1 malignant tumor was found, as well as 1 endometriosis cyst with hard atypia and 3 endometriosis without atypia, 2 of which were squamous metaplasia. All subjects in the sham group showed normal endometrial histology (Figure 1).

The sham (control) group shows normal endometrial histology. The endometrial autoimplantation group without DMBA induction showed similar histology to human tissue, with no identified atypia. Cysts covered by flat epithelium, cuboid to columnar, could be seen. Inflammatory cell infiltration was also found.

**Figure 2:**
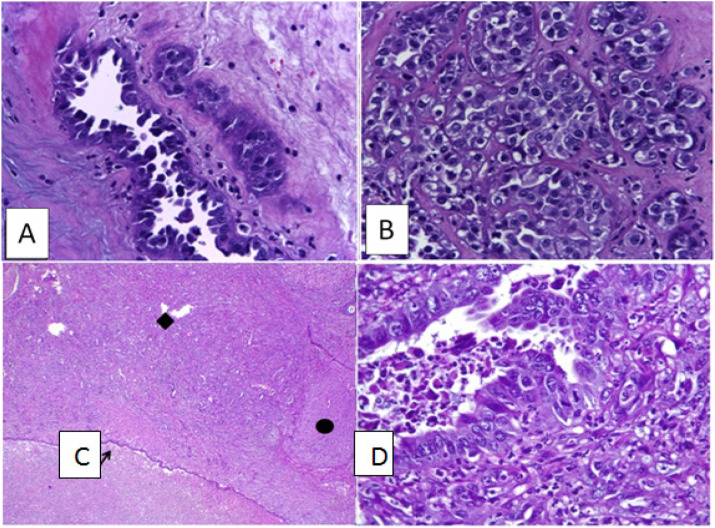
A-B. Clear cell carcinoma. A tubulocystic structure with a hobnail core and a solid structure resembling cobblestone can be found. Tumor cells appeared polygonal in shape with a pleomorphic nucleus and a pronounced nucleolus. The cytoplasm appears clear, mostly eosinophilic (HE400x). C. Endometrioid carcinoma. The tumor mass (♦) destructed the ovarium (●). The endometrial cyst wall was found (↑) (HE 40x). D. Endometrioid carcinoma forming a glandular structure. Tumor cells had round, oval, and columnar nuclei, with rough chromatin, which appeared vesicular with the nucleolus. Signs of mitosis were found. Cytoplasm, eosinophilic (HE400x).

The subjects in the endometrial autoimplantation group with 5-week DMBA induction all showed non-atypical endometriosis, one of which happened to be squamous metaplasia (Figure 1.A, B). The endometrial autoimplantation group with 10-week DMBA induction gave rise to non-atypical endometriosis in 3 rats, 2 of which were squamous metaplasia, 1 was atypical endometriosis (Figure 1.C, D), and 1 was clear cell carcinoma (Figure 2.A, B.).

The group of rats treated with a combination of endometrial autoimplantation and 20-week DMBA induction all had endometrioid carcinomas. Histologic features showed a tumor mass that formed a back-to-back glandular structure to a solid structure. The tumor cells had a moderate pleomorphic nucleus, rough chromatin, which appeared vesicular with the nucleolus. Cytoplasm appeared eosinophilic. Signs of mitosis were found (Figure 2.C, D).

*ARID1A expression*

**Figure 3:**
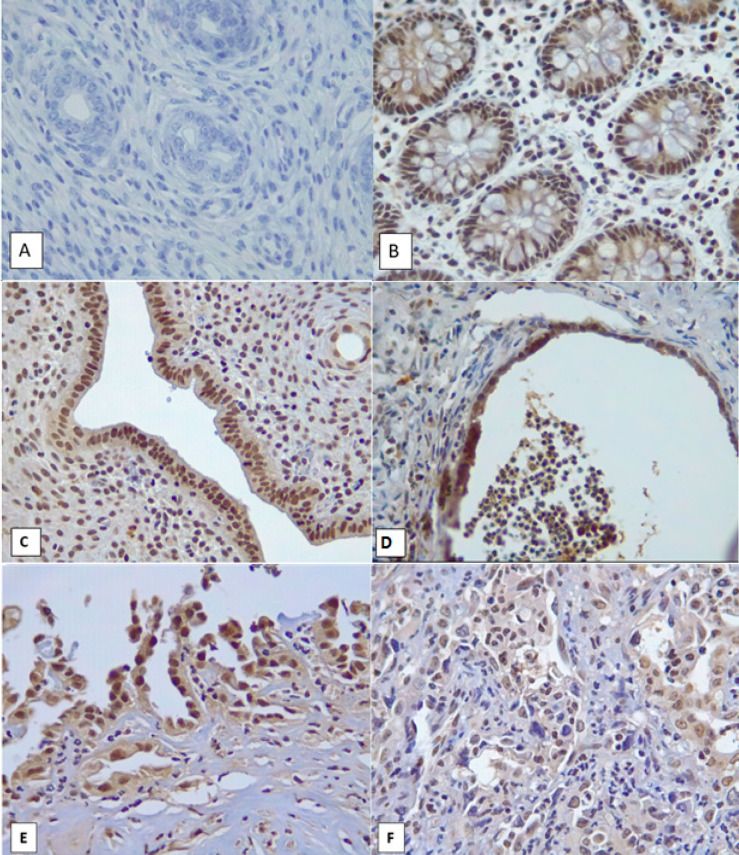
*ARID1A* Expression. A. Negative control. B. Positive control from intestinal tissue. C. ARID1A expression in normal endometrium in the sham group. D. ARID1A expression in non-atypical endometriosis. E. ARID1A expression in atypical endometriosis. F. Endometrioid carcinoma showed patchy negative ARID1A expression (A-E, HE 400x).

ARID1A staining (Figure 3) was assessed with Image J. *ARID1A* expression in the sham group was 98.5-100% with a median of 99.7%. *ARID1A* expression in the endometrial autoimplantation group yielded a range of value of 86-100% with a median of 95.10%.* ARID1A *expression in the endometrial autoimplantation group with 5-week DMBA induction was 94-100% with a median of 92.2%. *ARID1A* expression in the endometrial autoimplantation group with 10-week DMBA induction was 91.5-100% with a median of 92.2%.* ARID1A *expression in the endometrial autoimplantation group with 20-week DMBA induction was in the range of 71.5-100% with a median of 90.40 %. 

In the endometriosis group and groups with combination of autoimplantation and DMBA induction, the median percentage of *ARID1A *expression was lower compared to the sham group. However, the Kruskal-Walis test revealed that this difference was not significant (p=0.313). 

## Discussion

Spontaneous endometriosis cannot occur in rats. Rats do not experience the menstrual cycle but experience an estrus cycle. In the estrus cycle, there is no endometrial shedding, but resorption of the damaged endometrium. Implantation of the endometrium into the ovary is analogous to retrograde menstruation which can occur spontaneously in humans, causing endometrium cells to migrate to the ovary. In rats, this spontaneous process does not occur so that the process of transfer of endometrial tissue can be manipulated by autoimplantation techniques to the ovaries, where ectopic endometrial tissue can be obtained (Berkley et al., 2004; Grümmer, 2006; King et al., 2016). The combination of autoimplantation techniques and DMBA induction for 5 weeks resulted in non-atypical endometriosis, one of which was squamous metaplasia. Similar to the human condition, metaplastic lesions are often found in endometriosis.

Ovaries are likened to fertile soil for the growth of endometriosis cells. When ovulation occurs, the process of rupture and repetitive repairs cause local inflammation that changes the condition of the microenvironment. Cytokines and growth factors are produced at the site of ovarian rupture, producing chemotactic factors that attract cells outside the ovary, both benign and malignant cells. Post-ovulatory inflammation and pro-repair condition also function as tumorigenesis factors that support the transformation of malignancy and allow malignant cells to survive (Yang-Hartwich et al., 2014).

Endometriosis cysts in humans contain many iron elements which are thought to play a role in the process of transformation of endometriosis malignancies. The free-form or catalytic accumulation of iron mediates the formation of reactive oxygen species (ROS) through Fenton reactions and induces oxidative stress. Oxygen-free radicals produced from free iron induce mutations and DNA damage. This high iron content plays an important role in carcinogenesis originating from endometriosis (Yamaguchi et al., 2008).

Inflammation is considered as the hallmark of endometriosis. The high abnormal activity of intraperitoneal macrophages plays a role in the optimization of endometriosis growth by releasing angiogenic factors, therefore increasing the microvascularization of the parietal peritoneum. Endometrial implants in the ovary cause estrogen persistence and reduction of 2 progesterone receptor isoforms resulting in physiological milieu changes around the surface of the ovary. Progesterone can suppress cell proliferation and induce apoptosis. Changes in hormone receptors give rise to non-physiological hormonal conditions and can cause further progression towards malignancy (Yamaguchi et al., 2008). This is supported by the results of this study, which observed that the group with endometrial autoimplantation and 10-week DMBA induction obtained 1 (20%) atypical endometriosis. Atypical endometrosis is known to be an EAOC precursor lesion. In this group, there was also 1 (20%) malignancy with clear cell carcinoma. In the group observed until the 20th week, all malignancies were found with endometrioid carcinoma. Therefore, it can be drawn that time influences the emergence of malignant transformation in endometriosis.

In rats with endometrial implantation only, changes were also observed in the microenvironment. However, in these experimental animals, no iron accumulation was found. Components of ROS derived from iron degradation are not found in experimental animals like they are in humans. Therefore DMBA was used to induce malignancy. DMBA is an indirect carcinogen that will be metabolized by CYP1B1. DMBA metabolic results are DMBA-DE, which become DNA adducts and can induce mutation. CYP isoform is known to produce ROS during the metabolism of carcinogens through uncoupled reactions. ROS can enter diffusely into cells and interact with macromolecules such as lipids, proteins, and DNA to induce oxidative modification which eventually triggers the oncogenic process (Madden et al., 2014; Muqbil et al., 2006; Priyadarsini and Nagini, 2012).

*ARID1A *mutations are one of the most frequently reported molecular genetic changes in CCOC and EOC. *ARID1A *mutations cause loss of expression of the *BAF250a* protein, which normally suppresses cellular proliferation through the regulation of the transcription of p53-dependent suppressor tumors such as CDKN1A and SMAD3. ARID1A inactivation occurs early in the development of CCOC, or EOC (Ayhan et al., 2012). Borrelli et al., (2016) shows a partial loss of *ARID1A *expression in rectovaginal deep-infiltrating endometriosis (DIE), endometrioma, and in endometrial tissue used as controls. *ARID1A* mutations are also often found in endometriod carcinoma of the uterus (Gounaris et al., 2011).

EOC and CCOC in humans do not all experience *ARID1A* mutations. This is supported by observations of *ARID1A* expression that were not statistically significant but showed a tendency to decline in some tumors in the 20-week induction group. Wiegand et al., (2010) suggested that 46% of CCOC had an *ARID1A* mutation, whereas, in EOC, it was stated that 30% had an *ARID1A *mutation. EOC and CCOC without *ARID1A* mutations are referred to as wild-type ARID1A. However, tumors with *ARID1A* mutations can also show *ARID1A* expression, which is totally lost or partially lost. This missing *ARID1A *expression partly describes clonal mutations and the presence of tumor heterogeneity (Win et al., 2016).

The results of this study can complement current knowledge of *ARID1A* expression in the immunohistochemical profiles of DMBA-induced EAOC. EOC and CCOC can show mutations other than *ARID1A*. A research conducted by Salinah et al., (2018) revealed that DMBA-induced endometrioid carcinoma showed a decrease in p16INK4a protein expression.

In conclusion, histopathological changes occurred in rats given endometriosis autoimplantation and ovarian DMBA induction along with the emergence of malignant transformation in endometriosis. The combination of endometrial autoimplantation techniques and DMBA induction in the ovary produced atypical endometriosis, clear cell carcinoma, and endometrioid carcinoma. There was no significant difference in *ARID1A* expression between the treatment and control groups.
